# Proteomics Study of Peripheral Blood Mononuclear Cells in Down Syndrome Children

**DOI:** 10.3390/antiox9111112

**Published:** 2020-11-11

**Authors:** Chiara Lanzillotta, Viviana Greco, Diletta Valentini, Alberto Villani, Valentina Folgiero, Matteo Caforio, Franco Locatelli, Sara Pagnotta, Eugenio Barone, Andrea Urbani, Fabio Di Domenico, Marzia Perluigi

**Affiliations:** 1 Department of Biochemical Sciences “A. Rossi Fanelli”, Laboratory Affiliated to Istituto Pasteur Italia-Fondazione Cenci Bolognetti, Sapienza University of Rome, 00185 Rome, Italy; chiara.lanzillotta@uniroma1.it (C.L.); matteo.caforio@opbg.net (M.C.); sara.pagnotta@uniroma1.it (S.P.); eugenio.barone@uniroma1.it (E.B.); fabio.didomenico@uniroma1.it (F.D.D.); 2 Department of Basic Biotechnological Sciences, Intensivological and Perioperative Clinics, Università Cattolica del Sacro Cuore, 00168 Rome, Italy; viviana.greco@unicatt.it (V.G.); andrea.urbani@policlinicogemelli.it (A.U.); 3 Department of Laboratory Diagnostic and Infectious Diseases, Fondazione Policlinico Universitario Agostino Gemelli-IRCCS, 00168 Rome, Italy; 4 Pediatric and Infectious Disease Unit, Bambino Gesù Children’s Hospital, 00165 Rome, Italy; diletta.valentini@opbg.net (D.V.); alberto.villani@opbg.net (A.V.); 5 Department of Pediatric Hematology/Oncology and of Cell and Gene Therapy, Bambino Gesù Children’s Hospital, 00165 Rome, Italy; valentina.folgiero@opbg.net (V.F.); franco.locatelli@opbg.net (F.L.); 6 Department of Gynecology/Obstetrics and Pediatrics, Sapienza University of Rome, 00185 Rome, Italy

**Keywords:** proteomics, Down syndrome, peripheral blood mononuclear cells (PBMCs), unfolded protein response, oxidative stress

## Abstract

Down syndrome (DS) is the most common chromosomal disorder and the leading genetic cause of intellectual disability in humans, which results from the triplication of chromosome 21. To search for biomarkers for the early detection and exploration of the disease mechanisms, here, we investigated the protein expression signature of peripheral blood mononuclear cells (PBMCs) in DS children compared with healthy donors (HD) by using an in-depth label-free shotgun proteomics approach. Identified proteins are found associated with metabolic pathways, cellular trafficking, DNA structure, stress response, cytoskeleton network, and signaling pathways. The results showed that a well-defined number of dysregulated pathways retain a prominent role in mediating DS pathological features. Further, proteomics results are consistent with published study in DS and provide evidences that increased oxidative stress and the increased induction of stress related response, is a participant in DS pathology. In addition, the expression levels of some key proteins have been validated by Western blot analysis while protein carbonylation, as marker of protein oxidation, was investigated. The results of this study propose that PBMCs from DS children might be in an activated state where endoplasmic reticulum stress and increased production of radical species are one of the primary events contributing to multiple DS pathological features.

## 1. Introduction

Down syndrome (DS) is the most common chromosomal abnormality among live-born infants. The number of people with DS living in the United States has grown from 49,923 in 1950 to 206,366 in 2010 [1]. There are no population-based registries in Italy, but the prevalence of people with DS living is estimated at approximately 30,000. Life expectancy in children with DS has increased significantly over the past decade, but children with DS remain at higher risk of neonatal and infant mortality than children without DS, respectively (1.65% vs. 0.36 and 4% vs. 0.48%) [2]. Some of the most prominent features of the DS phenotype include mental retardation as well as an increased incidence of congenital heart disease, hypothyroidism, diabetes, leukemia and by the age of 40 they reported an increased risk of developing Alzheimer like dementia [3,4,5,6,7,8,9]. Furthermore, patients with DS show multiple defects in both numbers and function of innate and adaptive immunity [10,11]. Recent studies also stated that DS is associated with a primary defect of the B-cell compartment, characterized by a reduced number of IgM memory B and switched memory B [12,13]. Therefore, children with DS show a high susceptibility to recurrent infections, characterized by increased severity and a prolonged course [14]. No single gene or region of human chromosome 21 has been found to be responsible for all the common features of DS [15,16], therefore, it would be expected that multiple genes and other factors working in concert are responsible for the major DS phenotypes [4,5,6,7]. Consequently, most of the focus in the field has been focused to understand how the alterations in the expression of specific genes in Chr21 trisomic cells lead to developmental dysfunction and pathological manifestations [17]. Chronic oxidative stress (OS) and mitochondrial dysfunction are the key factors that are thought to contribute to additional clinical conditions observed in individuals with DS such as diabetes, immune system abnormalities, and autism spectrum disorder, as well as to the development of Alzheimer’s disease (AD) [18,19,20,21,22]. Increased OS has been linked to the triplication of the Cu/Zn superoxide dismutase (SOD), the transcription factor Ets-2, the stress-inducing factors DSCR1, and the amyloid precursor protein (APP), and it has been consistently observed in different DS primary cells including fibroblasts [20,23], cortical neurons, astrocytes, pancreatic cells, and lymphoblastic cell lines from both DS young and old patients [22,24,25].

The increase of OS results from increased basal levels of mitochondrial reactive oxygen species (ROS), deficits in electron transport chain components deficit in mitochondrial complex I, ATP synthase, ADP/ATP translocator, and adenylate kinase activities [23,26] from an adaptive downregulation of mitochondrial activity and from a reduction of antioxidant response [23,27,28]. Mitochondrial DNA mutations and alteration in mtDNA have also been reported in fibroblasts derived from people with DS and in DS brain tissue [29,30]. Furthermore, induced pluripotent stem cells (iPSCs) from people with DS and iPSCs-derived DS neurons show oxidative hallmarks and are more sensitive to oxidative damage than control cells [31,32]. Fragmented and bioenergetically inefficient mitochondria have also been observed in DS as a result of impaired MQC [33].

Recent studies on human and murine samples described a strong and complex crosstalk between altered protein homeostasis and OS in DS pathology [34,35,36,37,38]. Increased OS can target and aberrantly stimulate stress response pathways, thus promoting the dysregulation of protein homeostasis [35,39,40,41,42]. In the last years, the dysregulation of the unfolded protein response (UPR), as well as, the failure of autophagy and ubiquitin proteasome system (UPS) degradative pathways have been observed in DS and are emerging as a strong candidate mechanisms involved in the development of pathological conditions [43,44,45,46,47]. In addition, studies from Potier’s laboratory and others reported endosomal enlargement in peripheral and neuronal cells from DS cases supporting a role for early endosomal dysfunction and aberrantly regulated endosomal trafficking in the toxic events leading to DS pathology in the brain and in other organs [48,49,50,51,52]. An increasing number of studies also demonstrated that DS subjects are at high risk to develop either peripheral or brain metabolic defects, characterized by the dysregulation of the insulin signaling with reduced downstream pathways, and altered mitochondrial structure and function that, in turn, is associated with increased ROS production and OS [53].

In the present study, we performed a high-definition label-free shotgun proteomics analysis on PBMCs samples from DS and healthy children (healthy donors, HD) to identify novel molecular mechanisms in DS pathology and to investigate potential biomarkers based on the identified proteins in young diseased individuals. Results obtained from the comparative protein expression analysis confirmed that a well-defined number of dysregulated pathways, including stress responses, cellular trafficking, energy metabolism, and cell structure retain a prominent role in mediating DS pathological features.

## 2. Materials and Methods

### 2.1. Study Population

For this study, we recruited individuals referring from the Down Syndrome and Pediatric outpatient Clinic of Bambino Gesù Children’s Hospital in Rome. All the study participants underwent complete clinical workup including medical history collection, clinical examination, thropometric measurements and laboratory test. In Table 1 are listed all the clinical data of the subjects enrolled in the study including gender, age of participants, their body mass index calculated [BMI; weight (kg) × squared height (m^2^)], and comorbidities; for children under the age of 2 years old we did not calculate the BMI but the ratio between weight/height and the relative’s centile. The study was approved by the Ethical Committee of Bambino Gesù Children Hospital in Rome, Italy (protocol # 1771_OPBG_2019).

### 2.2. Samples Collection of Peripheral Blood Mononuclear Cells from HD and DS Subjects

Peripheral blood mononuclear cell (PBMC) was isolated from DS and healthy donors (HD) blood samples. For the isolation of PBMC, ACD-A-anticoagulated blood was centrifuged at 800× *g* for 30 min and the top layer containing the plasma was removed. The remaining blood was diluted with an equal volume of phosphate-buffered saline, pH 7.4 (PBS), containing 0.05 M ethylenediaminetetraacetic acid (EDTA; Invitrogen Corporation, Carlsbad, CA, USA). Total of 12.5 mL of diluted blood was layered over 25 mL of the Ficoll-Paque PLUS (GE Healthcare, Chicago, IL, USA). Gradients were centrifuged at 400× *g* for 30 min at room temperature in a swinging-bucket rotor without applying brake. The PBMC interface was prudently removed by pipetting and washed with PBS-EDTA by centrifugation at 250× *g* for 10 min. PBMC pellets were suspended in ammonium-chloride-potassium (ACK) lysing buffer (Invitrogen Corporation, Carlsbad, CA, USA) and incubated for 10 min at room temperature with gentle mixing to lyse the contaminating red blood cells (RBC), then washed with PBS-EDTA. Cell number and viability were determined using a countess automated cell counter (Invitrogen Corporation, Carlsbad, CA, USA). Non-viable cells were identified by staining with trypan blue, and cell viability was calculated using the total cell count and the count of non-viable cells. PBMCs were cryopreserved in liquid nitrogen in fetal calf serum (FCS; Invitrogen, Carlsbad, CA, USA) containing 10% dimethyl sulfoxide (DMSO; Thermo Fisher Scientific, Waltham, MA, USA) and stored until required for downstream analyses.

### 2.3. Protein Sample Preparation

The total protein extract from PBMCs was prepared in RIPA buffer (pH = 7.4) containing tris-HCl (50 mM pH = 7.4), NaCl (150 mM), 1% NP-40, 0.25% sodium deoxycholate, 1 mM EDTA, 0.1% SDS, supplemented with phosphatase and protease inhibitor (539132, Millipore, 1:100; P0044; Sigma-Aldrich, St. Louis, MO, USA; 1:100). Before clarification, the samples were sonicated on ice and then centrifuged at 16,000× rpm at 4 °C for 30 min to remove cellular debris. The supernatant was then used to determine the total protein concentration by the BCA method (Pierce, Rockford, IL, USA).

### 2.4. Protein Expression Analysis by nLC–HDMS^E^

Briefly, protein extracts derived from peripheral blood mononuclear cells (PBMCs) isolated from 6 DS subjects and 6 healthy donors (HD) blood samples were handled for enzymatic digestion according to the filter-aided sample preparation (FASP) protocol [54]. Briefly, as shown below the following steps were performed using filter tubes (Nanosep centrifugal device with Omega membrane-10 K MWCO): reduction (DTT 8 mM in urea buffer-8 M urea, and 100 mM Tris), alkylation (IAA 50 mM in urea buffer 8 M urea, and 100 mM Tris), and trypsin digestion (final trypsin concentration of 0.01 μg/μL). Label-free proteomic analysis was performed, as previously described by Greco V et al. [55] with few modifications. First, 300 fmol/μL of digested enolase from *Saccharomyces cerevisiae* (P00924) was added to each sample as an internal standard. Each digested sample (0.25 μg) was loaded onto a Symmetry C18 5 µm, 180 µm × 20 mm pre-column (Waters Corp., Milford, MA, USA), and was subsequently separated by a 120-min reversed-phase gradient at 300 nL/min (linear gradient, 2–40% ACN over 90 min) using a HSS T3 C18 1.8 μm, 75 μm × 150 mm nanoscale LC column (Waters Corp., Milford, MA, USA) maintained at 40 °C. Tryptic peptides were separated on an ACQUITY MClass System (Waters Corp., Milford, MA, USA) and then separated peptides were analyzed using a high-definition Synapt G2-Si mass spectrometer (Waters Corp., Milford, MA, USA) directly coupled to the chromatographic system. Differential protein expression was evaluated by a high-definition expression configuration mode (HDMSE), a data-independent acquisition (DIA) protocol where ion mobility separation (IMS) is integrated into LC-MSE workflow as described by Marini F. et al. [56]. The mass spectrometer parameters are set as: positive survey polarity of electrospray source (ES+), acquisition mode mass range 50–2000 *m/z*, capillary source voltage 3.2 kV, source T 80 °C, cone voltage 40 eV, TOF resolution power 20,000, precursor ion charge state 0.2–4, trap collision energy 4eV, transfer collision energy 2eV precursor MS scan time 0.5 sec, and fragment MS/MS scan time 1.0 sec. All spectra have been acquired in ion mobility separation mode (IMS) cycles with wave height at 40 V, wave velocity of 650 m/s, transfer wave height 4 V, and transfer wave velocity of 175 m/s. Data were post-acquisition lock mass corrected using the doubly charged monoisotopic ion of [Glu1]-Fibrinopeptide B (Waters Corp., Milford, MA, USA), sampled every 30 s. Each sample was run in four technical replicates. The analysis of differentially expressed proteins was performed according to Silva et al. [57] and Visser et al. [58]. Continuum LC-MS data from the four analytical replicates for each sample derived from both DS and HD PBMC were processed for qualitative and quantitative analysis using the ProteinLynx Global Server v3.0.3 software (PLGS, Waters Corp., Milford, MA, USA). The qualitative identification of proteins was obtained using the embedded ion accounting algorithm of the software PLGS and by searching against Homo Sapiens database (UniProt KB/Swiss-Prot Protein Knowledgebase restricted to homo sapiens taxonomy) to which the sequence from Saccharomyces cerevisiae Enolase (UniProtKB/Swiss-Prot AC: P00924) was appended. In order to obtain protein identifications, the PLGS software Search parameters include: automatic tolerance for precursor ions and for product ions, minimum 1 fragment ion matched per peptide, minimum 3 fragment ions matched per protein, minimum two peptide matched per protein, 2 missed cleavage, carbamydomethylation of cysteines and oxidation of methionine as fixed and variable modifications respectively. The identification of protein was based on the detection of more than two fragment ions per peptide, and more than two peptides measured per protein. False discovery rate (FDR) of the identification algorithm was set under 1%, based on a target decoy database. For quantitative expression analysis 300 fmol of Enolase has been set as calibration protein concentration. PLGS software uses the most reproducible proteotypic peptides for retention time and intensity of Enolase digestion (*m/z* 745.43, *m/z* 814.49, *m/z* 1288.70, *m/z* 1416.72, *m/z* 1578.80, and *m/z* 1840.89) to normalize the table of the exact mass on retention times (EMRTs). The expression analysis was performed considering two experimental groups, DS and HD, which include all the technical replicates derived from each sample (i.e., experimental condition, DS and HD: six biological samples × four technical replicates) following the hypothesis that each group was an independent variable (DS and HD). The differentially expressed proteins dataset was screened and filtered according to the following MS established criteria by considering only those identifications from the alternate scanning LC-HDMSE data exhibiting a good replication rate (at least three out of four runs of the same sample) and with *p* < 0.05 for the relative protein fold change (two-tailed Student’s *t* test). The significance of regulation level specified with a fold change of regulation higher than ±30%, which is typically 2–3 times higher than the estimated error on the intensity measurement, was used as a threshold to identify significant up- or down-regulation.

### 2.5. Bioinformatics and Network Analysis

To identify the biologically relevant molecular pathways, the proteomic datasets were analyzed using bioinformatic analysis tools based on QIAGEN’S Ingenuity Pathway Analysis (QIAGEN’S Ingenuity Pathway Analysis, Ingenuity Systems, http://www.qiagen.com/ingenuity) and STRING. Relevant functional associations have been explored. The analysis parameters were set as follows: direct and indirect relationships, endogenous chemical substances included all molecules, and/or relationships considered as the summary filter. The most significant categories associated with the loaded datasets were identified by calculating their significance (*p*-value, Fischer test). A *p*-value threshold was set at 0.05, which showed the probability of association between genes/proteins present in the datasets and each pathway (canonical pathway, and biological function).

### 2.6. Western Blot

For Western blot validations, 20 μg of protein from 8 DS subjects and 8 HD were resolved on Criterion TGX 4–15% 18-well (Tris-Glycine extended) Stain-Free precast gels (Bio-Rad, Hercules, CA, USA, #5678084) in Tris/Glycine/SDS (TGS) Running Buffer (Bio-Rad Laboratories, Hercules, CA, USA, # 1610772). After electrophoresis, the gel was placed on aChemi/UV/Stain-Free tray and then placed in a ChemiDoc MP imaging System (Bio-Rad Laboratories, Hercules, CA, USA, # 17001402) and UV-activated based with the stain-free gel settings to collect total protein load image. Stain-free imaging technology utilizes a trihalo compound to enhance natural protein fluorescence by covalently binding to tryptophan residues with a brief UV activation. Images of the gel or membrane after transfer were easily captured using stain-free gel settings. This allowed visualization, verification, and validation at all steps of electrophoresis and blotting. The stain-free technology allows for total lane normalization avoiding the use of housekeeping proteins (HKPs). Following electrophoresis and gel imaging, the proteins were transferred via the Trans Blot Turbo semi-dry blotting apparatus (Bio-Rad Laboratories, Hercules, CA, USA, # 1704150) onto nitrocellulose membranes (Bio-Rad, Hercules, CA, USA, # 162–0115). Membranes were blocked with 3% of BSA (bovine serum albumin 9048-46-8; SERVA Electrophoresis GmbH, Heidelberg, Germany) in 1X tris buffer saline (TBS; 1706435, Bio-Rad, Hercules, CA, USA) containing 0.01% Tween 20 and incubated overnight at 4 °C with the following primary antibodies: ATF6 (sc-166659; Santa Cruz Biotechnology; Dallas, TX, USA, 1:250), p^Ser724^IRE1α (NB100-2323; Novus Biologicals, Centennial, CO, USA; 1:1000), IRE1α (sc-390960; Santa Cruz Biotechnology, Dallas, TX, USA; 1:250), PRDX6 (A305-315A-M Bethyl, 1:1000), Gelsolin (sc-398244; Santa Cruz Biotechnology, Dallas, TX, USA; 1:500), and SOD-1 (sc-271014; Santa Cruz Biotechnology, Dallas, TX, USA; 1:500). Next day all membranes were washed three times with 1X tris buffer saline containing 0.01% Tween 20 and incubated for 1 h at room temperature with respective horseradish peroxidase-conjugated secondary antibodies: anti-rabbit (L005661; Bio-Rad Laboratories, Hercules, CA, USA; 1:20,000), anti-mouse (L005662; Bio-Rad Laboratories, Hercules, CA, USA; 1:20,000) from or anti-goat IgG (A5420; Sigma-Aldrich, St. Louis, MO, USA; 1:3000). Blots were then imaged via ChemiDoc MP imaging system using the Chemiluminescence settings. Subsequent determination of relative abundance via total protein normalization was calculated using Image Lab 6.1 software (Bio-rad Laboratories, Hercules, CA, USA).

### 2.7. Slot Blot Analysis and Protein Carbonylation

Protein carbonyls was used as a marker of protein oxidation and their levels were determined as described by Butterfield et al. [59]. Total of 5 μL from PBMCs samples was derivatized at room temperature for 20 min in 10 mM DNPH and 5 μL of 12% sodium dodecyl sulphate (SDS). Samples were than neutralized with 7.5 μL of neutralization solution (2 M tris in 30% glycerol). The derived samples (250 ng) were then blotted onto a nitrocellulose membrane under vacuum pressure using a slot-blot apparatus (Bio-Rad, Hercules, CA, USA). Membranes were blocked for 1 h at room temperature with 3% of BSA (bovine serum albumin 9048-46-8; SERVA Electrophoresis GmbH, Heidelberg, Germany) in 1X tris buffer saline (TBS; 1706435, Bio-Rad, Hercules, CA, USA) containing 0.01% Tween 20 and incubated for 2 h at room temperature with a 1:1000 dilution of rabbit polyclonal anti-DNP primary antibody. Then, membranes were washed three times with 1X tris buffer saline solution containing 0.01% Tween 20 (T-TBS) and incubated for 1 h at room temperature with the respective alkaline phosphatase secondary antibodies from Sigma-Aldrich (1:10,000 dilution of anti-rabbit IgG alkaline phosphatase). The membranes were later washed three times in (T-TBS) and developed with a solution of nitro-tetrazolium blue chloride (0.2 mM) and 5-bromo-4-chloro-3-indolyl phosphate dipotassium (0.4 mM) in ALP buffer (0.1 M Tris, 0.1 M NaCl, 5 mM MgCl2; pH 9.5). Blots were dried, acquired with Chemi-Doc MP imaging system Bio-Rad Laboratories, Hercules, CA, USA, # 17001402) (Bio-Rad, Hercules, CA, USA) and analyzed using Image Lab 6.0 software (Bio-Rad, Hercules, CA, USA).

To have an internal control we use samples with no primary antibody or samples pre-treated with NaBH4 to reduce protein carbonyls and resulted in no staining.

### 2.8. Statistical Analysis

For proteomics analysis six samples were employed for each group. As previously described [44], sample size was calculated using G-Power 3.1 software using the following parameters: Effect size = 2.5; Err- prob = 0.05; Power = 0.95; sample size of *n* = 6 for each group with an actual power of 0.97. For Western blot analysis, we selected eight samples per group (G-power parameters are: Effect size = 2; Err- prob = 0.05; Power = 0.95; sample size of *n* = 8 for each group with an actual power of 0.96). The t-test was used to evaluate differences between HD and DS where p values equal * *p* = 0.05, ** *p* = 0.01. Data are expressed as mean ± SEM per group. Values above or below two standard deviations of the mean were considered outliers and discarded from the data set. All statistical analyses were performed using Graph Pad Prism 8.0 software (GraphPad, La Jolla, CA, USA). As reported in the dedicated section, mass spectrometry raw data have been analyzed according to the well-established parameters for DIA-MS^E^ acquisition [57,58].

## 3. Results

### 3.1. Proteomics Analysis

In the present study a comparative characterization of PBMC proteomes from 6 DS and 6 HD cases was performed to investigate the putative changes in protein expression that could allow to gain insight into the molecular mechanisms of DS pathology. The differential protein expression analysis was carried out by a HD-MS^E^ isotope label-free profiling. Quality control measures were performed on the analytical replicates to ensure the reproducibility of the mass measurement and chromatographic retention time of each peptide. We acknowledge a limitation for sample size in the present study, however data obtained were consistent with previous analysis and have been partially confirmed by specific immunochemical analysis. As thoroughly described in Section 2.3, applying the statistical parameters on the identified proteins (such as fold change of regulation higher than ±30%), 178 proteins have been shown as statistically differentially expressed across both conditions.

**Figure 1:**
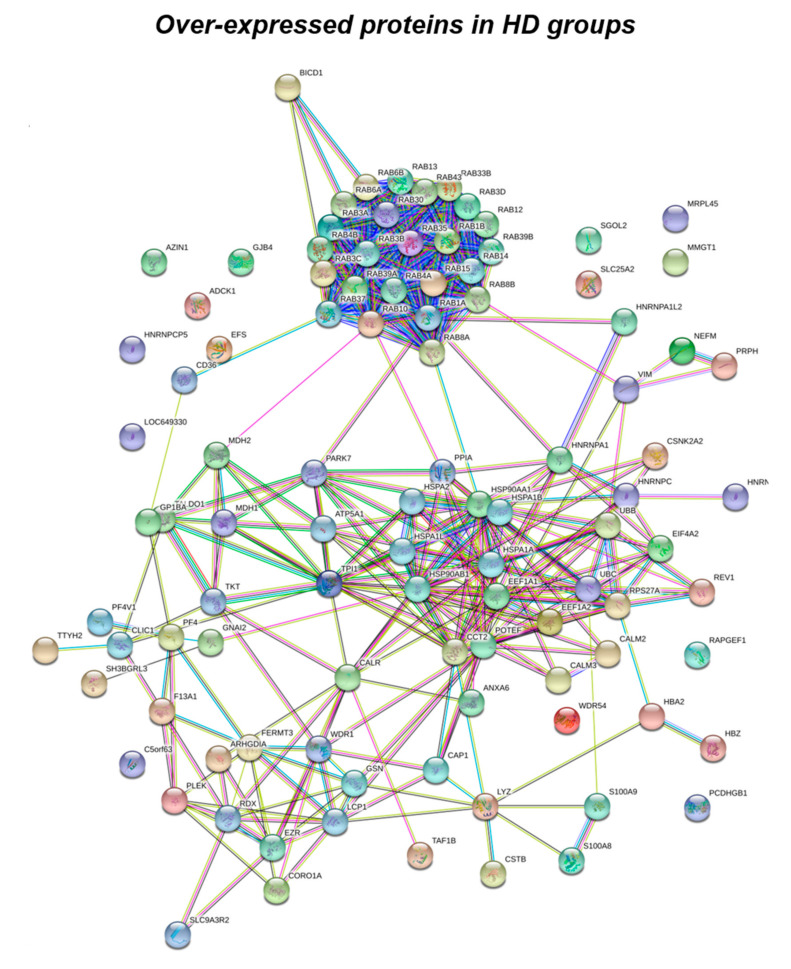
Functional network enrichment in the HD group.

**Figure 2:**
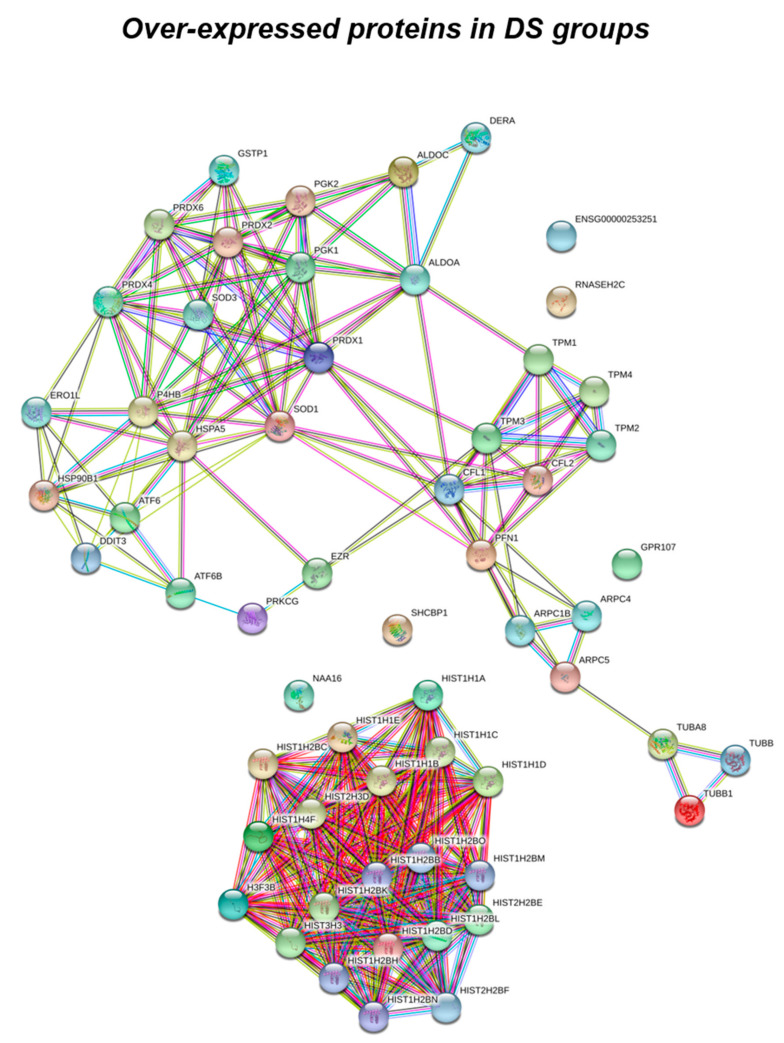
Functional network enrichment in the DS group.

Data obtained from label-free proteomics analysis and then bioinformatics-based speculations show that all the proteins, differently expressed in the experimental groups, clustered in a well-defined number of functions including stress response, trafficking, metabolism, DNA structure, cytoskeleton network and signaling, as represented in Figure 1 and Figure 2, and as listed in Table 2, Table 3 and Table 4.

**Figure 3:**
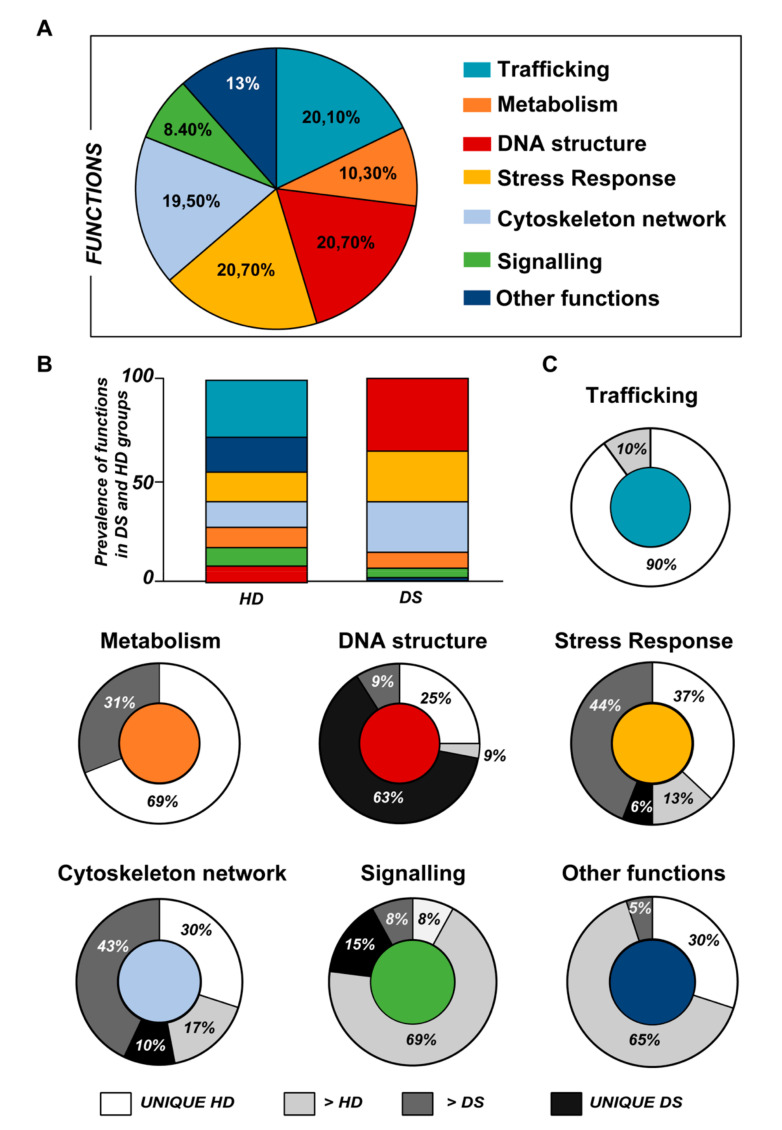
(**A**) Pie chart representing all the proteins grouped according to their function. (**B**) Representative graph showing the prevalence distribution of the listed functions in the HD and DS groups, respectively. (**C**) Pie chart representing all the functions reported in the work and the related alterations among the groups of analysis (UNIQUE HD, UNIQUE DS, >HD, and >DS).

According to the analytical features of the mass spectrometer used and the results from quantitative expression analysis, the identified proteins were then clustered as follows: proteins with a different expression comparing both DS and HD conditions (over-expressed or down-expressed, with fold change >30%), and highly expressed proteins, so-called “unique” proteins, whose compared expression ratio is more than ten times higher so that they can be considered present in only one specific group. Thus, we identified four groups of comparison: UNIQUE HD that include proteins overexpressed in HD with a fold >10; overexpressed in HD (>HD) that include protein with a fold of increase >1.3 but <10; UNIQUE DS that include proteins overexpressed in DS with a fold >10; overexpressed in DS (>DS) that include protein with a fold of increase >1.3 but <10. Intriguingly, the HD groups include proteins belonging to all six functional groups but demonstrate a high prevalence of trafficking proteins (28%) as also evident in Figure 1 and Table 2. We report also a 15% of stress response proteins, a 13% of cytoskeletal proteins, a 10% of metabolic proteins, a 9% of proteins related to signaling network, and an 8% of DNA structure proteins. In addition, the 17% of proteins with increased expression in HD groups do not fall in any of the functional networks taken into considerations. The DS groups display an altered expression for proteins mainly involved in DNA structure (36%) and stress response (25%) as evident in Figure 2 and Figure 3B, while other functional groups that presents overexpressed protein in DS are: cytoskeleton network for the 25%, metabolic pathways for the 8%, cell signaling for the 5%, and a broad array of function for the remaining 2% (Figure 3B).

Subsequently, by analyzing for each identified functional set, the percentage and the identity of the proteins belonging to UNIQUE HD, >HD, UNIQUE DS, and >DS groups, we sought to delineate the pathways that are mainly affected during DS pathology, their role in disease progression and their potential value as biomarkers. The 100% of all the proteins included in intracellular trafficking group are over increased in HD groups with the 90% and 10% belonging to UNIQUE HD and to >HD respectively (Figure 1 and Figure 3C); in detail as listed in Table 1 we identified 25 proteins belonging to the Rab family, a member of the Ras superfamily of small G proteins. In the cell metabolism group, UNIQUE HD proteins account for the 69%, while the 31% belongs to >DS (Figure 3C). Pathway analysis revealed that these proteins are related to energy metabolism, glucose metabolism, ornithin metabolic pathway, and urea cycle. In detail, the protein linked to energy metabolism are: cytoplasmatic and mitochondrial malate dehydrogenase, lactate dehydrogenase, ATP synthase subunit alpha mitochondrial, glyceraldehyde-3-phosphate dehydrogenase, fructose-bisphosphate aldolase A/C, phosphoglycerate kinase 1, 2, transaldolase, transketolase. Protein linked to the ornithin metabolic pathway and urea cycle are the Antizyme inhibitor 1 and the mitochondrial ornithine transporter 2. Analyzing the stress response proteins network, it is evident that the 37% of the proteins are UNIQUE HD, the 13% are >HD, the 6% belong to UNIQUE in DS, and the 44% are overexpressed in DS (Figure 2 and Figure 3C). Some notable proteins include those involved in the response to ER stress, protein folding in the ER (ERO1 and PDI), ER-nucleus signaling pathway (ATF6, ATF6-alpha, B and CHOP), and in cellular chaperoning, such as GRP78 (Table 1). Focusing on ER stress, endoplasmin is a molecular chaperone that functions in the processing and transport of secreted proteins, and functions in endoplasmic reticulum-associated degradation (ERAD). Intriguingly, all the above-mentioned proteins, associated with ER stress, were found to be over-expressed in DS compared to HD, thus confirming the involvement of increased UPR in the pathophysiology of Down syndrome. Furthermore, other relevant over-expressed proteins in DS involved in stress response with antioxidant properties are SOD-1, peroxiredoxin 1, 2, 4, 6, and glutathione S-transferase. Regarding the cytoskeleton network, statistical analysis reveals that these proteins fall into the HD groups for the 47% (specifically, 30% UNIQUE in HD and 17% >HD) and into the DS group for the 53% (specifically, 10% are UNIQUE DS, and 43% are overexpressed in DS). The UNIQUE HD group include the following proteins: Gelsolin, Annexin A6, Calmodulin-1, 2, 3, WD repeat-containing protein 1, 54, Vimentin, Protocadherin gamma, Plastin-2, Adenylyl cyclase-associated protein 1, and Fermitin family homolog 3. The only protein unique in DS cases is actin-related protein 2/3 complex subunit, while the proteins over-expressed in DS are: Cofilin-1, 2, Tropomyosin alpha and beta chain, Tubulin alpha and beta with different isoforms, Myosin-9, Ezrin, and Profilin-1. An altered expression of the proteins involved in the DNA structure strongly affects the DS groups, indeed, only the 28% of protein belongs to the HD groups including the heterogeneous nuclear ribonucleoprotein A1 and heterogeneous nuclear ribonucleoprotein C1/C2, while 72% of the identified protein is part of both the UNIQUE DS and >DS groups. In detail, five types of histone proteins, H1, H2A, H2B, H3, and H4, have been identified as increased in DS (Figure 2). The proteins involved in signaling network group represents a small amount of the total identified proteins (8%) and comprises: Pleckstrin OS, Platelet glycoprotein 4, Platelet glycoprotein Ib alpha chain, SHC SH2 domain-binding protein 1, Protein kinase C gamma, and GPR107. The distribution of these proteins between the groups of comparisons revealed that the 77% belong to the HD groups (specifically, 8% UNIQUE HD and 69% > HD) and the 27% to DS groups (respectively, 15% to UNIQUE DS and 8% to the >DS). At final we identified 20 proteins (13% of the total), which do not fall in any of the above-mentioned networks and may hold various biological functions, have been identified in HD groups (95%).

### 3.2. Western Blot Analysis

**Figure 4:**
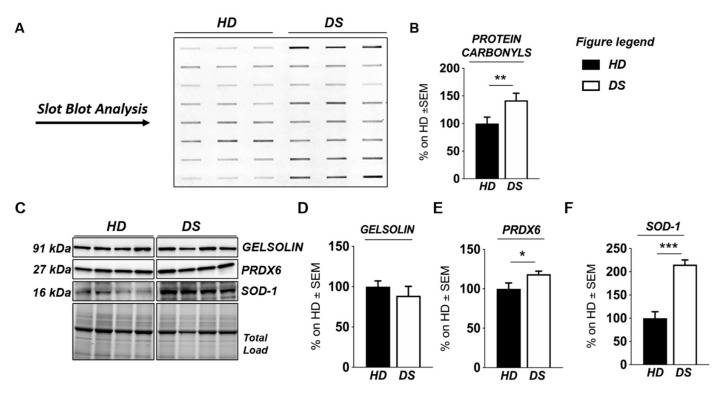
Human PBMCs from DS individuals show the induction of antioxidant responses to counteract OS. Slot blot analysis of total protein carbonylation. Panel (**A**) (left hand side): Slot blot of representative samples from healthy donors (HD) and Down syndrome (DS) groups. A triplicate of eight samples per group is showed. Panel (**B**) (right hand side): Densitometric analysis of total protein carbonylation in healthy donors (HD) and Down syndrome (DS) groups. (**C**) Representative Western blot showing SOD-1, PRDX6, and GELSOLIN in PBMCs from DS and HD. (**D**) Quantification of panel C showing levels of total GELSOLIN. (**E**) Quantification of panel C showing levels of PRDX6 (**E**) Quantification of panel C showing levels of total GELSOLIN. (**F**) Quantification of panel (C) showing levels of SOD1. Densitometric values shown in the bar graph are the mean of six samples for each group normalized for total load and are given as percentage of HD, set as 100%. Statistical significance was determined using Student t-test analysis (* *p* < 0.05, ** *p* < 0.01, *** *p* < 0.001).

**Figure 5:**
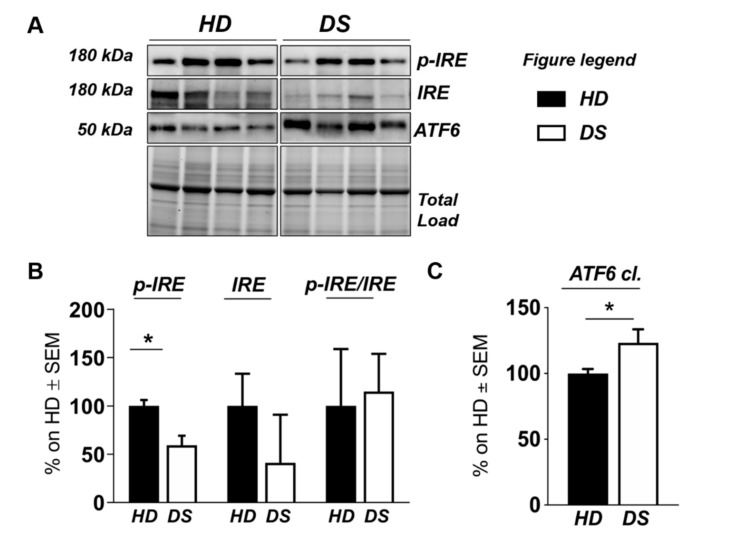
Human PBMCs from DS individuals show selective activation of the unfolded protein response. (**A**) Representative Western blot showing ATF6, p-IRE1, and IRE1 in PBMCs from DS and HD. (**B**) Quantification of panel (A) showing levels of total ATF6. (**C**) Quantification of panel (A) showing levels of pIRE1/IRE1 ratio. Densitometric values shown in the bar graph are the mean of eight samples for each group normalized for total load and are given as percentage of HD, set as 100%. Statistical significance was determined using Student *t*-test analysis (* *p* < 0.05).

To validate the results obtained with label-free proteomics analysis and to strengthen the notion that the over induction of UPR and of OS detoxifying mechanisms occur in DS for of the increase of the OS environment, we analyzed, by slot blot, protein carbonylation as a surrogate index for total protein oxidation and, by Western blot, the proteins involved in ER stress and OS responses (Figure 4 and Figure 5).

We found a significant increase of protein carbonylation in DS PBMCs compared to HD. As can be seen from Figure 4B, the global protein carbonylation load was higher in DS group with respect to HD group (+42%, *p* = 0.02). The elevation of protein carbonylation parallel with the increase of SOD-1, triplicated in DS, whose overexpression is observed both by mass spectrometry-based proteomics and WB (+115.4%; *p* < 0.001) (Figure 4F). As a further validation of the MS data, the overexpression of PRDX-6 in DS was also confirmed by WB analysis (+18%; *p* = 0.03) and support the induction of antioxidant responses to counteract OS in DS PBMCs (Figure 4E). In addition, by WB analysis we analyzed the expression levels of Gelsolin, a protein linked to cytoskeletal network, but we did not find a significant increase in DS as expected (Figure 4D). Moreover, proteomics data supports the increase of UPR components in DS PBMCs. Previous studies by us have already demonstrated the increased expression of GRP78 (BiP), CHOP, and of the PERK pathway in human PBMCs from DS cases [45]. To add additional insights into the UPR induction in DS periphery, we performed the analysis of ATF6 and of IRE1. We show a significant increase of ATF6 cleaved form in DS PBMCs compared with HD (+23.3, *p* = 0,04) (Figure 5C) confirming MS-proteomics data, while the analysis of IRE1 demonstrates a decrease of protein expression but no alterations concerning its phosphorylation state (Figure 5B), strengthen the idea of the induction of selected UPR branches in DS pathology.

## 4. Discussion

In the present study, a comparative proteomic investigation was performed to identify the putative protein biomarkers of DS that could allow to get a deeper comprehension of the complex mechanisms responsible for DS pathological phenotypes. Gene dosage is believed to play a significant role in determining the wide variability of DS phenotypes [15,60,61,62]. While gene dosage may contribute to the phenotype associated with DS, an exact mechanism and specific gene network underlying DS abnormalities are yet to be elucidated [63,64]. Differentially expressed proteins were identified and a comprehensive study on the proteins associated with DS imbalance was carried out in PBMCs isolated from young DS individuals compared with heathy age-matched donors. PBMCs, by reflecting at systemic level cellular alterations driven by trisomic condition, represent a valuable model to elucidate the molecular mechanisms of DS pathology, including those that characterize neurodegeneration [43,45]. Collected results agree with published studies from our group and others showing the early alterations of specific cellular pathways in DS individuals that are considered to contribute to the accelerated-aging phenotype, ultimately resulting in the early onset of Alzheimer dementia in DS [5,34,35,36,49,65,66,67,68,69].

We suggest that a well-defined number of pathways, including intracellular trafficking, stress responses, cytoskeleton network, and energy metabolism are significantly disrupted in PBMCs from young DS individuals, reflecting alterations already observed in other tissues [5,35,53]. The presence of Rab GTPases, increasingly expressed in HD cases only, demonstrate a massive dysfunction of the intracellular membrane trafficking in DS. Alterations of Rab GTPases, or of the membrane compartments that they regulate, are associated with virtually all cellular activities in health and disease. Rab GTPases were initially discovered in brain tissue, where their abundance and functional adaptations reflect neuronal challenges for membrane trafficking [70,71]. However, Rabs are found in all eukaryotic cells, where they mediate fundamental processes of vesicle sorting and transport between target membranes [72,73]. Consequently, Rab GTPases are commonly used as markers and identifiers of various organelles and vesicles in the endocytic and secretory systems. Of particular interest in the present study is RAb-3A, whose downregulation was already reported in human frontal cortex from DS individuals [74]. Further, in a previous work, Reddy et al. have shown a substantial loss of presynaptic vesicle proteins and postsynaptic proteins, including Rab 3A, in brains from AD patients compared to controls subjects supporting a role of this protein in brain neuropathology [75]. In this scenario, our data suggest that the reduction of Rab3A could be an early event in DS that occurs at different body compartments thus representing a shared mechanism of cell alteration. Furthermore, in the present study we found both rab10 and rab2 as UNIQUE proteins in HD cases. Rab1 and Rab2 regulate the transport of vesicles between ER and Golgi [76]. Amber R. English et al. have also identified Rab10 as an ER-specific Rab GTPase that regulates ER structure and dynamics [77]. They show that Rab10 localizes to the ER and to dynamic ER-associated structures that track along microtubules and mark the position of new ER tubule growth. Rab10 depletion or expression of a Rab10 GDP-locked mutant alters the ER morphology, resulting in fewer ER tubules. Another major finding of this study was that the protein levels of Rab GDP-dissociation inhibitor 2 (GDI-2) were significantly decreased in DS, as observed in a fetal Down syndrome brain by R. Weitzdoerfer et al. [78]. Rab GDP-dissociation inhibitor 2 (GDI-2) is a regulatory protein involved in membrane release of Rabs [79] that holds an essential role in vesicle formation, vesicle docking, and membrane fusion [80]. The depletion of the *GDI* gene in yeast led to various transport defects in the cell, demonstrating the high importance of GDIs function [81]. Furthermore D’ Adamo et al. showed that GDI has an important role in neuronal function and a mutation in the gene encoding GDI-1 is responsible for X-linked mental retardation [81]. Taken together these results suggest that decreased levels of GDI-2, together with the alteration of Rabs proteins, found in PBMCs from DS patients may represent one of the multiple factors leading to impaired vesicles transport observed in DS [48].

Membrane trafficking is essential for protein synthesis, processing, sorting, and turnover in the ER and Golgi apparatus. The ER is a multifunctional organelle that coordinates protein folding, lipid biosynthesis, and calcium storage and release [82]. The alteration of ER trafficking is associated with the perturbations of ER homeostasis that lead to ER stress and to the activation of specific stress responses involved in protein folding and/or degradation. These response mechanisms include the induction of the UPR and of re-folding proteins such as PDIs. Our proteomics analysis demonstrates the differential expression, in PBMCs from DS children, of proteins belonging to ER stress responses with a role in protein folding and unfolded protein binding. We found the altered expression of GRP78, ATF6, and CHOP components of the UPR, and of ERO1 and PDIA1. The UPR is composed of three main branches including the PKR (dsRNA-dependent protein kinase)-like ER kinase (PERK) branch, the inositol-requiring enzyme 1 (IRE-1) branch, and the activating transcription factor 6 (ATF6) branch. Under basal conditions, these specialized ER membrane-associated sensor proteins are bound by the ER chaperone GRP78 (also known as BiP) and are maintained in an inactive state [82,83]. Accumulation of unfolded/misfolded/mutated proteins in the ER lumen activates adaptive UPR mechanisms through release of GRP78 from the sensor proteins and the initiation of specific cellular responses for the restoration of ER homeostasis [82]. Once the ER stress is over activated, protein synthesis overtakes protein-folding capacity, then ATF6 translocates to the Golgi where it is cleaved [84,85,86]. The ensuing ATF6 fragment (pATF6(N)) translocates to the nucleus and initiates the expression of its target genes such as chaperones, genes involved in ERAD and pXBP1(S), and also of the pro-apoptotic gene CHOP [87]. Therefore, CHOP plays an important role in the switch between pro-survival and pro-apoptotic responses and [88,89] its regulation is central to adjust the sensitivity of cells to ER stress. Here, we suggest that ATF6 plays a crucial part in the dynamics of CHOP induction, where perturbation of ATF6 led to slightly increased CHOP levels. The crucial role of ATF6 in CHOP dynamics during the induction of the UPR has been recently published by Yang et al. [90]. Further, a recent study from our laboratory demonstrated the increased expression of GRP78 and CHOP along with the over induction of PERK and eIF2α in DS PBMCs and human brain supporting the concept of the putative role of aberrant UPR induction in promoting DS pathology. Interestingly, our current data confirm that ER stress and UPR are primary events in DS and might have a prominent role in pathological processes [45]. Such hypothesis is further corroborated by different authors that observed dysfunctional UPR and ISR in DS human and mice, supporting a role for trisomy-related aberrant UPR/ISR induction in the disruption of the proteostasis network [43,47]. Over the above-cited proteins involved in the UPR and folding related to the ER, the selective recognition of oxidized/misfolded proteins by molecular chaperones (HSPs) is the first step toward their elimination. Heat shock protein 70 (HSP70) and heat shock protein 90 (HSP90) are chaperones that interact with the outer mitochondrial membrane, stabilizing the unfolded state of the nascent proteins and thereby preventing their aggregation. We showed that HSP70 and HSP90 were significantly downregulated in DS suggesting a prevention in the formation of the appropriate interactions with the proteins target resulting in protein misfolding and a consequent exacerbation of oxidative stress. The increase of oxidative stress is demonstrated to occur in our sample and is known to be involved in DS phenotype as an effect of the increased production of pro-oxidant species due to the triplication of *SOD-1* gene, among other. However, an involvement of differentially induced antioxidant responses has been also documented. We previously observed in DS blood-derived and brain samples the depletion of Nrf2-related antioxidant response, as an effect of Bach1 triplication, and its uncoupling with UPR defining a further degree of connection between proteostasis and OS [45]. Here we show that the increased expression levels of peroxiredoxins subtypes (Prx-proteins) in DS PBMCs suggest the activation of the Prx system due to unbalanced redox homeostasis [91]. Overall, the increased expression of Prx subtypes was already observed in brain patients with AD and DS [92].

Histones proteins represent a common target of ROS and RNS irreversible damage, that is able to alter their folding, expression, and stability, as well as, to induce their aberrant post-translational modification [93] thus severely impacting the global structure of chromatin, gene expression, genome stability, and replication. The alteration of proteins involved in DNA structure found by proteomic data concern mainly the DS group. We identified the altered expression of all the five types of histone proteins: H2A, H2B, H3, H4, and H1; and, except for H4, they all were identified as multiple isoforms. The increase in histone transcript levels that normally occurs during aging suggest a protection of the cells from premature aging, while the reduced histone expression in the short-lived mutants is a cause of their shortened lifespan. In agreement, Feser et al. demonstrated that the increase in the gene expression of all four core histones extended the median lifespan of asf1 mutants by 65% [94]. Within this context, our data suggest that the increased histones expression might represent a response to counteract the accelerated aging observed in DS.

Proteomics data also highlight the alteration of a central cellular function, the energy production. Collected results suggest that young DS individuals show an altered metabolic profile as indicated by a reduced expression of mitochondrial enzymes and of enzymes belonging to the pentose phosphate pathway (PPP), while in the presence of increased expression of glycolytic enzymes, including aldolase, GAPDH, and PGK1,2. Glycolysis is a fundamental feature of all cells and is therefore involved in a range of cellular responses that have been associated with both neurodevelopmental and neurodegenerative disorders. This includes effects on the immune system, cytoskeletal abnormalities, synaptic plasticity, and neurogenesis. We suggest that upregulation of glycolysis may be a compensatory mechanism in response to impaired mitochondrial function. Interestingly, among the glycolytic enzyme, fructose bisphosphate aldolase that catalyzes the reversible cleavage of F1,6PP to two triose phosphates, both of which continue through glycolysis, occupies a central position in glycolysis and gluconeogenesis pathways. Aldolase has been shown to boost glycolysis upon phosphoinositide 3-kinase (PI3K)/Akt signaling [95]. When glycolysis is activated, GAPDH has been reported to become a rate-limiting pathway step. Interestingly, GAPDH is often the most highly concentrated protein in glycolysis suggesting that the role of this high expression is to support the increased amount of glycolytic flux [96]. However, beside the above evidences support a condition of activated glycolysis, several published studies demonstrate that DS exhibit mitochondrial defects [20,22,26], ultimately responsible of reduced ATP production. Further, a reduction of transaldolase and transketolase levels, both involved in the second phase of PPP, may suggest decreased ability of the cell to replenish the cycle thus ultimately affecting both levels of energy substrates as well as reducing equivalents in the form of NADPH. Abnormalities in glucose metabolism and the link to metabolic syndrome in DS patients have been recently proposed [53] with evidences deriving from metabolome profile of plasma from DS [97,98], genes associated with glycolysis and signs of abnormal glucose metabolism evidenced in DS individuals and mouse models thereof [53]. Taken together, this signature suggests that a condition of hypometabolism occurs in DS individuals, likely because of the lowered glucose uptake, an early occurrence of insulin resistance associated with reduced mitochondrial activity [67]. Interestingly, our group, highlighted for the first time that markers of brain insulin resistance are evident in DS brain even before the development of AD pathology [67], suggesting that these alterations might support the mechanisms associated with intellectual disability, as well as the early onset of AD in people with DS [66].

Increasing evidences indicates that cytoskeletal abnormalities are observed already in prenatal life and may be largely responsible for the cortical dysgenesis in DS [65]. Among cytoskeleton components, we found that actin-related protein 2/3 complexes, tropomyosin, tubulin, cofilin, and myosin are overexpressed in DS vs. healthy controls. The microtubule cytoskeleton network is made up of tubulin subunits and actin filaments and serves multiple roles in neurons [99]. It provides a structural framework for axons and dendrites, representing a major determinant of neuronal size and morphology. It also serves as a track for transport and plays essential roles in growth and development. In contrast to microtubules that function individually, actin filaments work in networks or bundles that function to control cell shape, distribution of membrane proteins, and cell–cell interactions. Interestingly, in DS, cytoskeleton integrity seems to be strictly related to aberrant expression of Dyrk1A [100]. Published studies showed that both brain tissue and immortalized lymphocytes of DS patients displayed a significant reduction in the yield of all the major cytoskeletal proteins co-immunoprecipitated with DYRK1A antibodies [101]. Similarly to DS cells, overexpression of DYRK1A in trisomic TgDYRK1A mice was shown to cause alterations in actin dynamic through increased stability of actin filaments [102]. Further, results from Ori-McKenney et al. demonstrated that the regulation of microtubule dynamics by DYRK1A-mediated phosphorylation is critical for dendritic patterning and neuronal function, revealing a previously unidentified mode of post-translational microtubule regulation in neurons and uncovering a conserved pathway for a DS-associated kinase [100]. Conversely, Weitzdoerfer et al. reported the reduction of actin-related protein complex 2/3 in a fetal Down syndrome brain [103]. Our findings showing altered expression of cytoskeleton proteins in DS vs. healthy controls suggest that immune peripheral cell also retains the similar aberrant phenotype that is likely to play a central role in neurons. Dysfunction of cytoskeleton network may be considered a key pathological signature of DS.

Finally, we found the aberrant expression of several signaling proteins among which the reduced expression of protein kinase C (PKC) in DS group results in particular interest for the comprehension of the pathological processes. PKC represents a family of proteins including ten different members that represent ~2% of the entire kinome and display an almost ubiquitous expression throughout the human body. PKC enzymes are activated by signals such as increases with the concentration of diacylglycerol (DAG) or calcium ions (Ca_2_^+^) [104]. Once active PKCs are involved in controlling the function of other proteins through the phosphorylation of hydroxyl groups of serine and threonine amino acid residues. Reduced PKC activity was demonstrated in the brain of a DS mouse model [105] and in agreement with this, we support the decreased functionality of PKC signaling as pathological contributor of DS phenotype.

## 5. Conclusions

In summary, we found that PBMCs from young DS individuals recapitulate cellular defects that are considered to play a prominent role in DS pathological phenotypes. In detail, we identified proteins involved in metabolic pathways, cellular trafficking, stress response, cytoskeleton network, and cell signaling. In particular, collected data suggest that among the above-mentioned pathways increased OS and the over induction of stress-related response may confer to young DS individuals an activated state that may become a fertile ground that favors the accumulation, over lifespan, of multiple cellular damages, ultimately leading to different pathological outcomes including the onset of Alzheimer-like dementia. In addition, our data support the idea of employing blood cells as a model to complement the understanding of the key pathological mechanisms of DS. Further, considering that blood cells can be easily collected from living patients, they offer the unique opportunity for human studies aimed at identifying biomarkers of the disease, as well as testing novel therapeutic strategies.

## Abbreviations


**Aβ**
amyloid beta peptide
**AD**
Alzheimer disease
**AOR**
antioxidant response
**APP**
amyloid precursor protein
**ARE**
antioxidant response element
**ATF6**
activating transcription factor 6
**ATP**
adenosine triphosphate
**BiP-GRP78**
Binding immunoglobulin—Glucose regulated protein 78
**CHOP (DDTI3)**
DNA damage-inducible transcript 3
**Chr21**
chromosome 21
**CN**
central nervous system
**DS**
Down syndrome
**Dyrk1A**
dual specificity tyrosine phosphorylation regulated kinase 1A
**eIF2α**
eukaryotic initiation factor 2 alpha
**ER**
endoplasmic reticulum
**GAPDH**
glyceraldehyde 3 phosphate dehydrogenase
**H1A**
histone 1A
**HSP**
heat shock protein
**IRE1**
inositol-requiring enzyme 1
**ISR**
integrated stress response
**NADPH**
nicotinamide adenine dinucleotide phosphate
**Nrf2**
nuclear factor erythroid 2-related factor 2
**OS**
oxidative stress
**PBMC**
peripheral blood mononuclear cell
**PERK**
protein kinase R (PKR)-like endoplasmic reticulum kinase
**PGK**
phosphoglycerate kinase
**PPP**
pentose phosphate pathway
**PKC**
protein kinase C
**PRDX**
peroxiredoxin

## Figures and Tables

**Table 1:** Clinical characteristics of Down syndrome (DS) and healthy donors (HD) individuals.

Subject	Diagnosis	Age	Sex	BMI	Centile	Comorbidities
**HD 1**	Healthy donors	2	F	n/a	96	Asthma
**HD 2**	Healthy donors	14	F	19.8	54	Rash
**HD 3**	Healthy donors	9	F	17.6	68	Abdominal pain
**HD 4**	Healthy donors	4	M	15.7	54	Kawasaki disease
**HD 5**	Healthy donors	8	F	20	93	Headache
**HD 6**	Healthy donors	3	M	16.2	49	Kawasaki disease
**HD 7**	Healthy donors	12	M	19.6	54	Abdominal pain
**HD 8**	Healthy donors	7	F	20	92	Headache
**DS 1**	Down Syndrome	6	M	14.9	9	Behavioral trouble
**DS 2**	Down Syndrome	4	M	20.6	40	CAV surgery, behavioral trouble
**DS 3**	Down Syndrome	5	M	17.5	64	sleep apnea
**DS 4**	Down Syndrome	17	F	24.5	35	leukopenia
**DS 5**	Down Syndrome	1	F	21.9	48	hypothyroidism
**DS 6**	Down Syndrome	1	F	n/a	0	prematurity
**DS 7**	Down Syndrome	1	F	n/a	31	FPIES
**DS 8**	Down Syndrome	3	F	16.1	35	FPIES

**Table 2:** Differentially expressed proteins in DS and HD groups identified using HD-MS^E^ label-free mass spectrometry analysis clustered in the related molecular networks. (1) Experimental group in which proteins are mainly expressed; (2) protein name; (3) accession number according to UniProtKB/Swiss-Prot Protein Knowledgebase; (4) protein found highly represented (defined as unique) in DS or HD PBMCs protein extracts; (5) ratio of expression between DS and HS according to quantitative expression analysis by PLGS 3.03.

(1) Group	(2) Protein Description	(3) Uni Prot Accession Number	(4) Highly Expressed	(5) DS/HD Ratio
***Metabolism***				
**Unique HD**	L-lactate dehydrogenase A, B, C	P00338; P07195; P07864		
	Malate dehydrogenase_cytoplasmic	P40925	HD	
	Malate dehydrogenase_mitochondrial	P40926	HD	
	ATP synthase subunit alpha_mitochondrial	P25705	HD	
	Transaldolase	P37837	HD	
	Transketolase	P29401	HD	
	Triosephosphate isomerase	P60174	HD	
	Antizyme inhibitor 1	O14977	HD	
	Mitochondrial ornithine transporter 2	Q9BXI2	HD	
**Over-expressed in HD**	N/A	N/A	N/A	N/A
**Unique DS**	N/A	N/A	N/A	N/A
**Over-expressed in DS**	Glyceraldehyde-3-phosphate dehydrogenase	P04406		5.2
	Fructose-bisphosphate aldolase A, C	P04075; P09972		3.17
	Phosphoglycerate kinase 1, 2	P00558; P07205		1.86
***Trafficking***				
**Unique HD**	Rab1 (A, B, C)	P62820; Q9H0U4; Q92928	HD	
	Rab3 (A, B, C, D)	P20336; P20337; Q96E17; O95716	HD	
	Rab4 (A, B)	P20338; P61018	HD	
	Rab6 (A, B)	P20340; Q9NRW1	HD	
	Rab8 (A, B)	P61006; Q92930	HD	
	Rab10	P61026	HD	
	Rab12	Q6IQ22	HD	
	Rab13	P51153	HD	
	Rab14	P61106	HD	
	Rab15	P59190	HD	
	Rab30	Q15771	HD	
	Rab33 (B)	Q9H082	HD	
	Rab35	Q15286	HD	
	Rab37	Q96AX2	HD	
	Rab39 (A, B)	Q14964; Q96DA2	HD	
	Rab43	Q86YS6	HD	
	Peripherin	P41219	HD	
	Chloride intracellular channel protein 1	O00299	HD	
	AarF domain-containing protein kinase 1	Q86TW2	HD	
**Over-expressed in HD**	Rho GDP-dissociation inhibitor 1	P52565		0.12
	Protein bicaudal D homolog 1	Q96G01		0.15
	Membrane magnesium transporter	Q8N4V1		0.3
**Unique DS**	N/A	N/A	N/A	
**Over-expressed in DS**	N/A	N/A		N/A
***DNA Structure***				
**Unique HD**	Heterogeneous nuclear ribonucleoprotein A1	P09651	HD	
	Heterogeneous nuclear ribonucleoprotein A1-like 2	Q32P51	HD	
	Heterogeneous nuclear ribonucleoprotein C-like 1, 2, 3, 4	O6081; B2RXH8; B7ZW38; P0DMR1	HD	
	Heterogeneous nuclear ribonucleoproteins C1/C2	P07910	HD	
	TATA box-binding protein-associated factor RNA polymerase I subunit B	Q53T94	HD	
**Over-expressed in HD**	Putative male-specific lethal-3 protein-like 2	P0C860		0.56
**Unique DS**	Histone H1.1,2, 3,4,5	Q02539; P16403; P16402; P10412; P16401	DS	
	Histone H2B type 1-B, C, D, H, K, L, M, N, O	P33778; P62807; P58876; Q93079; O60814; Q99880; Q99879; Q99877; P23527	DS	
	Histone H2B type 2- E, F	Q16778; Q5QNW6	DS	
	Histone H2B type 3	Q8N257	DS	
	Histone H3.1,2,3	Q16695; Q71DI3; P84243	DS	
**Over-expressed in DS**	Shieldin complex subunit 3	Q6ZNX1		1.78
	Ribonuclease H2 subunit C	Q8TDP1		2.4
	Histone H4	P62805		2.51
***Stress Response***				
**Unique HD**	Poliubiquitin B, C	P0CG47; P0CG48	HD	
	Heat shock 70 kDa protein 1A, 1B	P0DMV8; P0DMV9	HD	
	Heat shock 70 kDa protein 1, 2	P34931; P54652	HD	
	Heat shock protein HSP 90-alpha, beta	P07900; P08238	HD	
	Ubiquitin-40S ribosomal protein S27a	P62979	HD	
	Calreticulin	P27797	HD	
	Parkinson disease protein 7	Q99497	HD	
	Glutaredoxin-like protein C5orf63	A6NC05	HD	
**Over-expressed in HD**	T-complex protein 1 subunit beta	P78371		0.3
	Protein S100-A8, A9	P05109; P06702		0.26
	Peptidyl-prolyl cis-trans isomerase A	P62937		0.4
**Unique DS**	Nitric oxide synthase-interacting protein	Q9Y314	DS	
	Glutathione S-transferase	P09211	DS	
**Over-expressed in DS**	Cyclic AMP-dependent transcription factor ATF-6 alpha and beta	P18850; Q99941		2.2
	DNA damage-inducible transcript 3 protein (CHOP)	P35638		3.4
	Endoplasmic reticulum chaperone BiP (GRP78)	P11021		2.07
	ERO1-like protein alpha	Q96HE7		3.04
	Protein disulfide-isomerase (PDI)	P07237		8.7
	Peroxiredoxin-1, 2, 4, 6	Q06830; P32119; Q13162; P30041		2.88; 3.7; 4.2; 4.8
	Endoplasmin	P14625		2.4
	Superoxide dismutase [Cu-Zn]	P00441		6.7
	Extracellular superoxide dismutase [Cu-Zn]	P08294		5.4
***Cytoskeleton Network***				
**Unique HD**	Gelsolin	P06396	HD	
	Annexin A6	P08133	HD	
	Calmodulin-1, 2, 3	P0DP23; P0DP24; P0DP25	HD	
	Protocadherin gamma	Q9Y5G3	HD	
	Neurofilament medium polypeptide	P07197	HD	
	Plastin-2	P13796	HD	
	Adenylyl cyclase-associated protein 1	Q01518	HD	
**Over-expressed in HD**	POTE ankyrin domain family member F	A5A3E0		0.6
	Radixin	P35241; P15311		0.7
	Na(+)/H(+) exchange regulatory cofactor NHE-RF2	Q15599		0.13
	Vimentin	P08670		0.8
**Unique DS**	Actin-related protein 2/3 complex subunit 1, 4, 5	O15143; P59998; O15511	DS	
**Over-expressed in DS**	Cofilin-1, 2	P23528; Q9Y281		1.67; 1.7
	Tropomyosin alpha-1,3, 4 chain	P09493; P06753; P67936,		2.21; 2.18; 2.46
	Tropomyosin beta chain	P07951		2.46
	TUBA4B Putative tubulin-like protein alpha-4B Iso 1	Q9H853		2.43
	TUBA8 Tubulin alpha-8 chain Iso 2	Q9NY65		5.13
	TUBB1 Tubulin beta-1 chain Iso 1	Q9H4B7		4.28
	TUBB2B Tubulin beta-2B chain Iso 1	Q9BVA1		3.8
	Myosin-9	P35579		7.29
	Ezrin	P15311		2.8
	Profilin-1	P07737		7.4
***Signaling***				
**Unique HD**	Guanine nucleotide-binding protein G(i) subunit alpha-2	P04899	HD	
**Over-expressed in HD**	Pleckstrin	P08567		0.53
	Platelet factor 4	P02776; P10720		0.24
	Platelet glycoprotein Ib alpha chain	P07359		0.22
	Platelet glycoprotein 4	P16671		0.26
	Embryonal Fyn-associated substrate	O43281		0.32
	SH3 domain-binding glutamic acid-rich-like protein	Q9H299		0.2
	Gap junction beta-4 protein	Q9NTQ9		0.4
	Casein kinase II subunit alpha	P19784		0.37
**Unique DS**	SHC SH2 domain-binding protein 1	Q8NEM2	DS	
	Protein kinase C gamma	P05129	DS	
**Overexpressed in DS**	GPR107	Q5VW38		8.4
***Other Functions***				
**Unique HD**	WD repeat-containing protein 1, 54	O75083; Q9H977	HD	
	Cystatin-B	P04080	HD	
	Putative elongation factor 1-alpha-like 3	Q5VTE0	HD	
	Coagulation factor XIII A chain	P00488	HD	
	Coronin-1A	P31146	HD	
**Over-expressed in HD**	Hemoglobin subunit alpha, zeta	P69905; P02008		0.64; 0.45
	Protein tweety homolog 2	Q9BSA4		0.4
	Elongation factor 1-alpha 1	P68104; Q5VTE0; Q05639		0.09
	Eukaryotic initiation factor 4A-II	Q14240		0.2
	Lysozyme C	P61626		0.39
	Shugoshin 2	Q562F6		0.46
	Fermitin family homolog 3	Q86UX7; Q13905; Q9UBZ9		0.34; 0.46; 0.6
	39S ribosomal protein L45_ mitochondrial	Q9BRJ2		0.14
**Unique DS**	N/A	N/A	N/A	
**Overexpressed in DS**	N-alpha-acetyltransferase	Q6N069		2.21

**Table 3:** Highlights of the five most significant reactome pathways involving proteins overexpressed in the HD group.

Reactome Pathways in HD			
Pathway	Description	Count in Gene Set	False Discovery Rate
**HSA-8873719**	RAB geranylgeranylation	23 of 63	3.35 × 10^−31^
**HSA-9007101**	Rab regulation of trafficking	16 of 118	2.57 × 10^−15^
**HSA-8876198**	RAB GEFs exchange GTP for GDP on RABs	14 of 86	2.18 × 10^−14^
**HSA-5653656**	Vesicle-mediated transport	26 of 64	6.66 × 10^−14^
**HSA-199991**	Membrane Trafficking	23 of 612	1.27 × 10^−11^

**Table 4:** Highlights of the 5 most significant reactome pathways involving proteins overexpressed in the DS group.

Reactome Pathways in DS			
Pathway	Description	Count in Gene Set	False Discovery Rate
**HSA-2262752**	Cellular responses to stress	27 of 384	1.17 × 10^−27^
**HSA-2559586**	Cellular responses to external stimuli	27 of 459	5.50 × 10^−26^
**HSA-2559586**	DNA Damage Stress Induced Senescence	15 of 61	3.98 × 10^−22^
**HSA-2559583**	Cellular Senescence	15 of 161	1.70 × 10^−16^
**HSA-195258**	RHO GTPase effectors	17 of 273	3.41 × 10^−16^
